# Supplementary figures and images for: Neuronal-specific methylome and hydroxymethylome analysis reveal significant loci associated with alcohol use disorder
Source: Front Genet. 2024 Apr 3;15:1345410. doi: 10.3389/fgene.2024.1345410 (PMC11021708; doi:10.3389/fgene.2024.1345410)

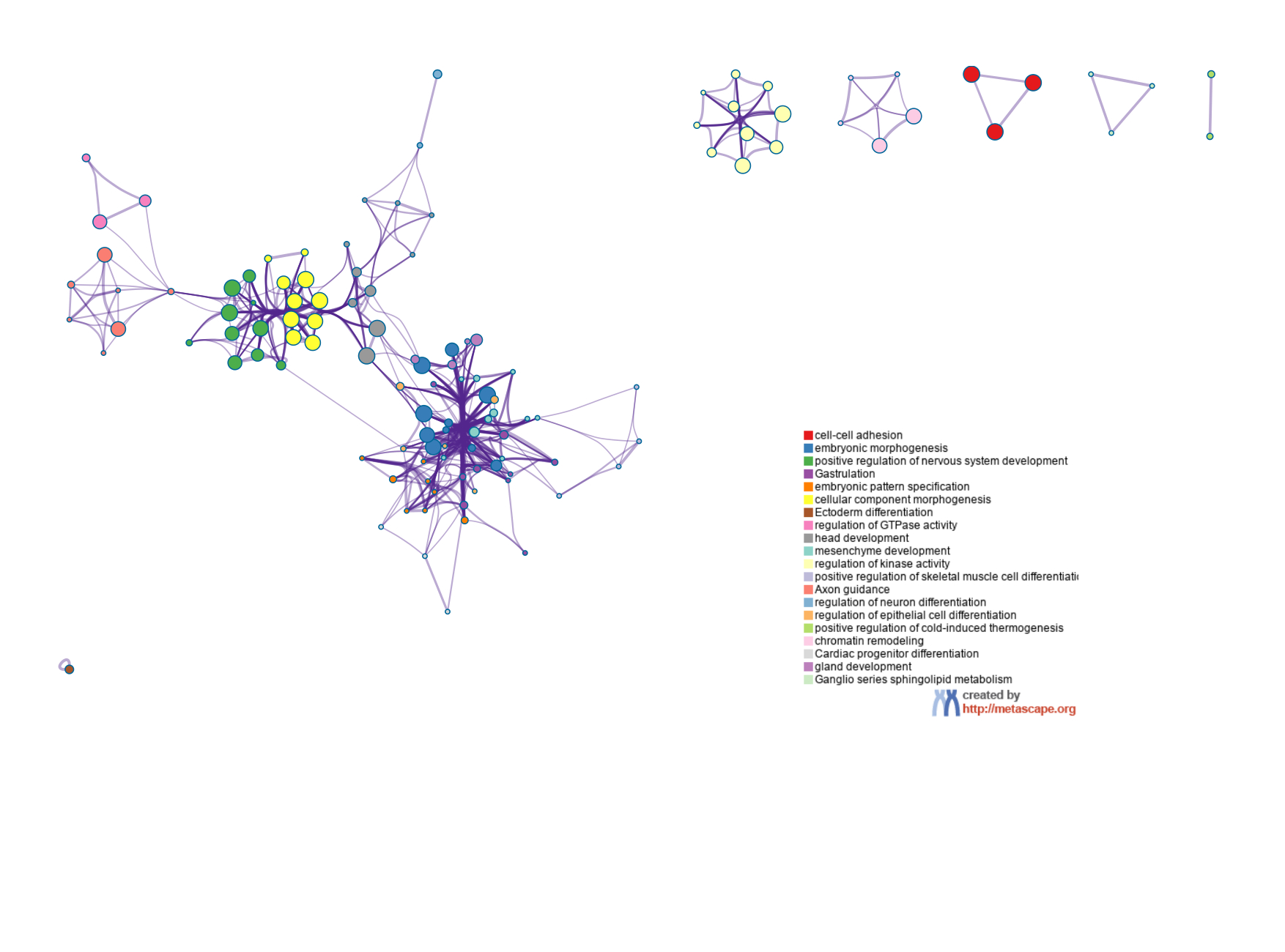

Supplement: Supplementary file 1 [file Image3.JPEG]

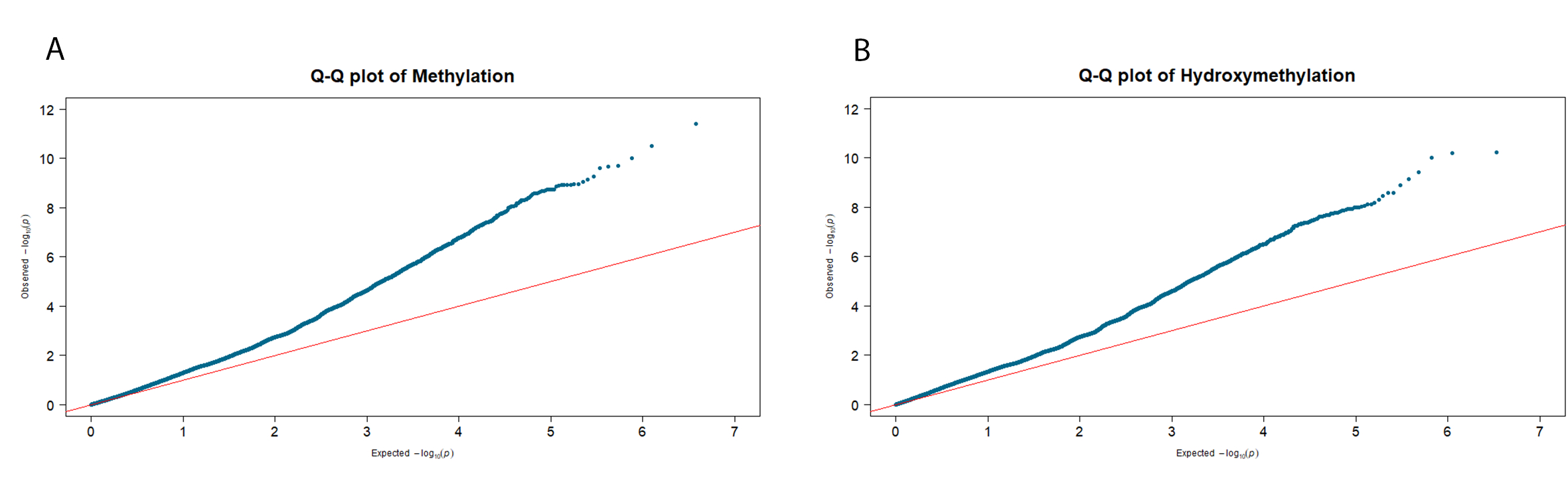

Supplement: Supplementary file 2 [file Image1.JPEG]

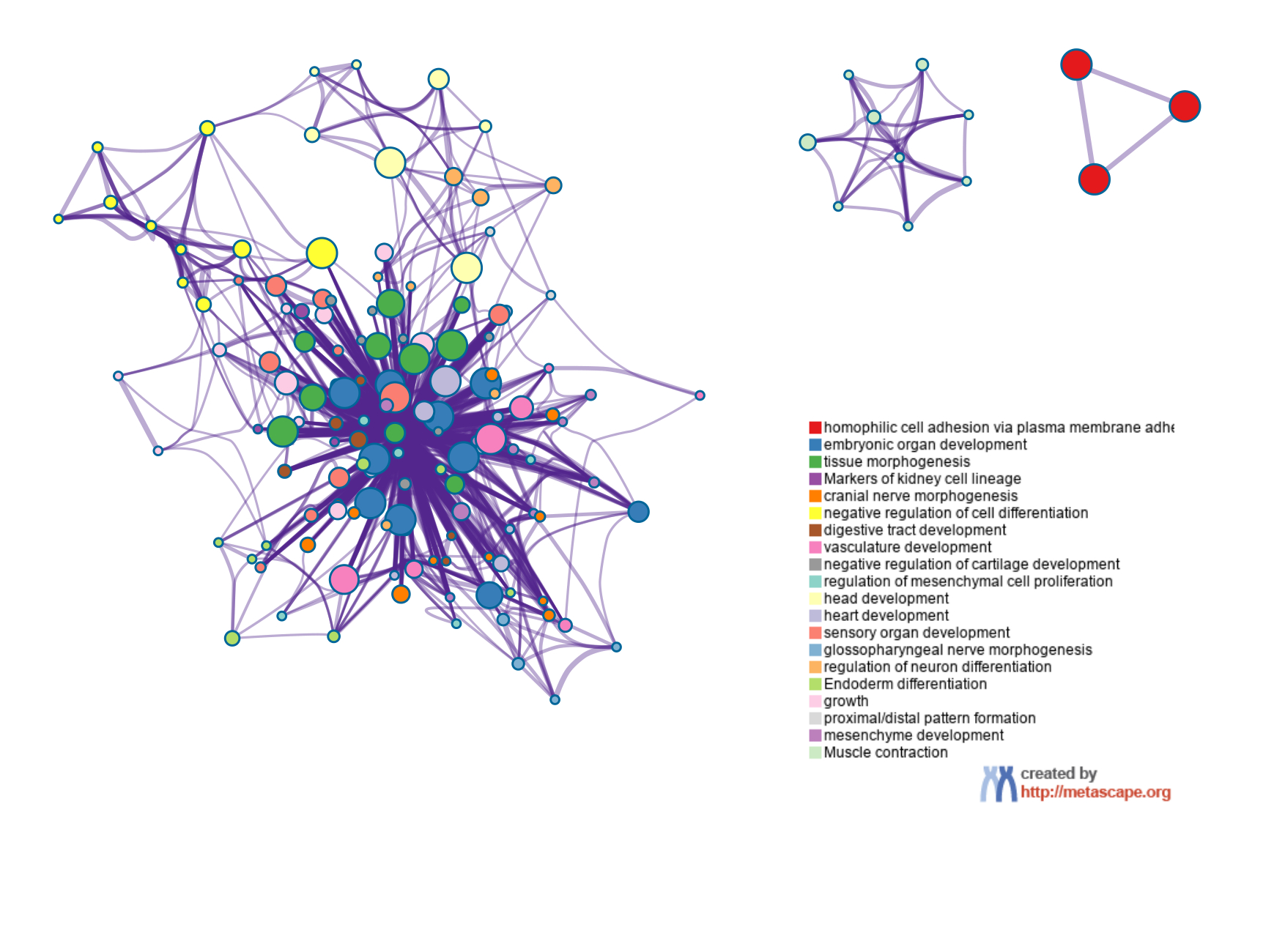

Supplement: Supplementary file 3 [file Image4.JPEG]

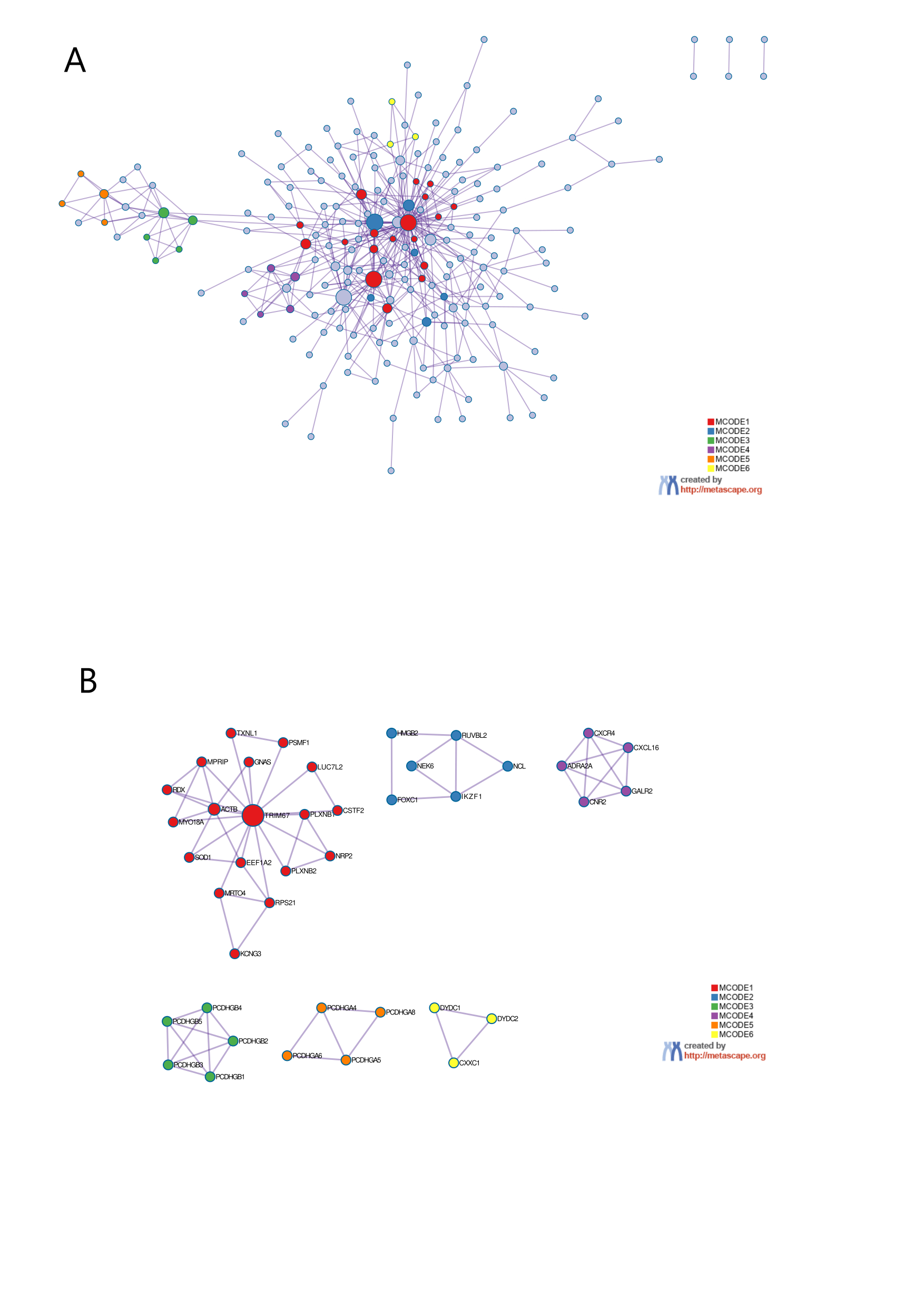

Supplement: Supplementary file 4 [file Image7.JPEG]

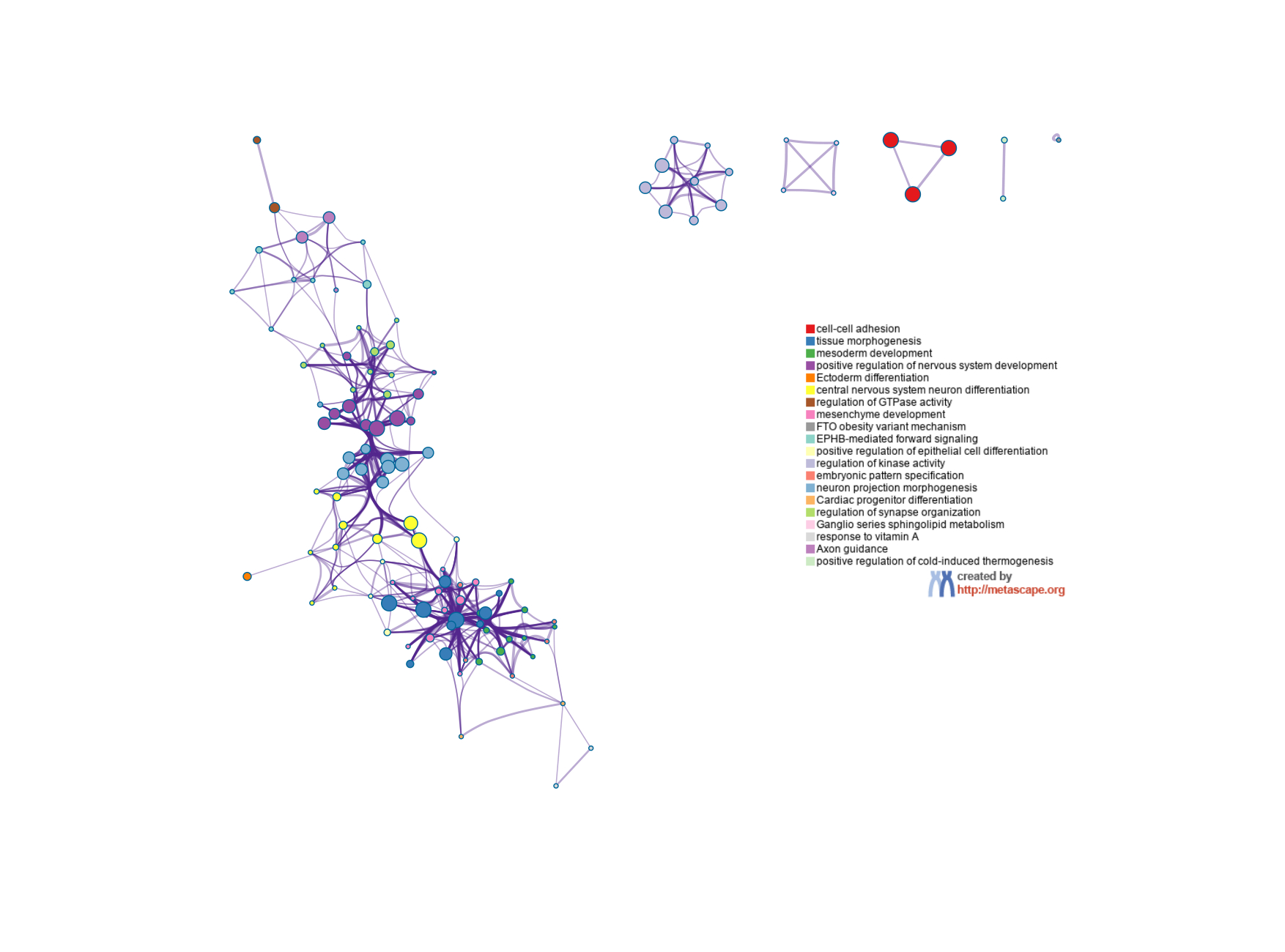

Supplement: Supplementary file 5 [file Image2.JPEG]

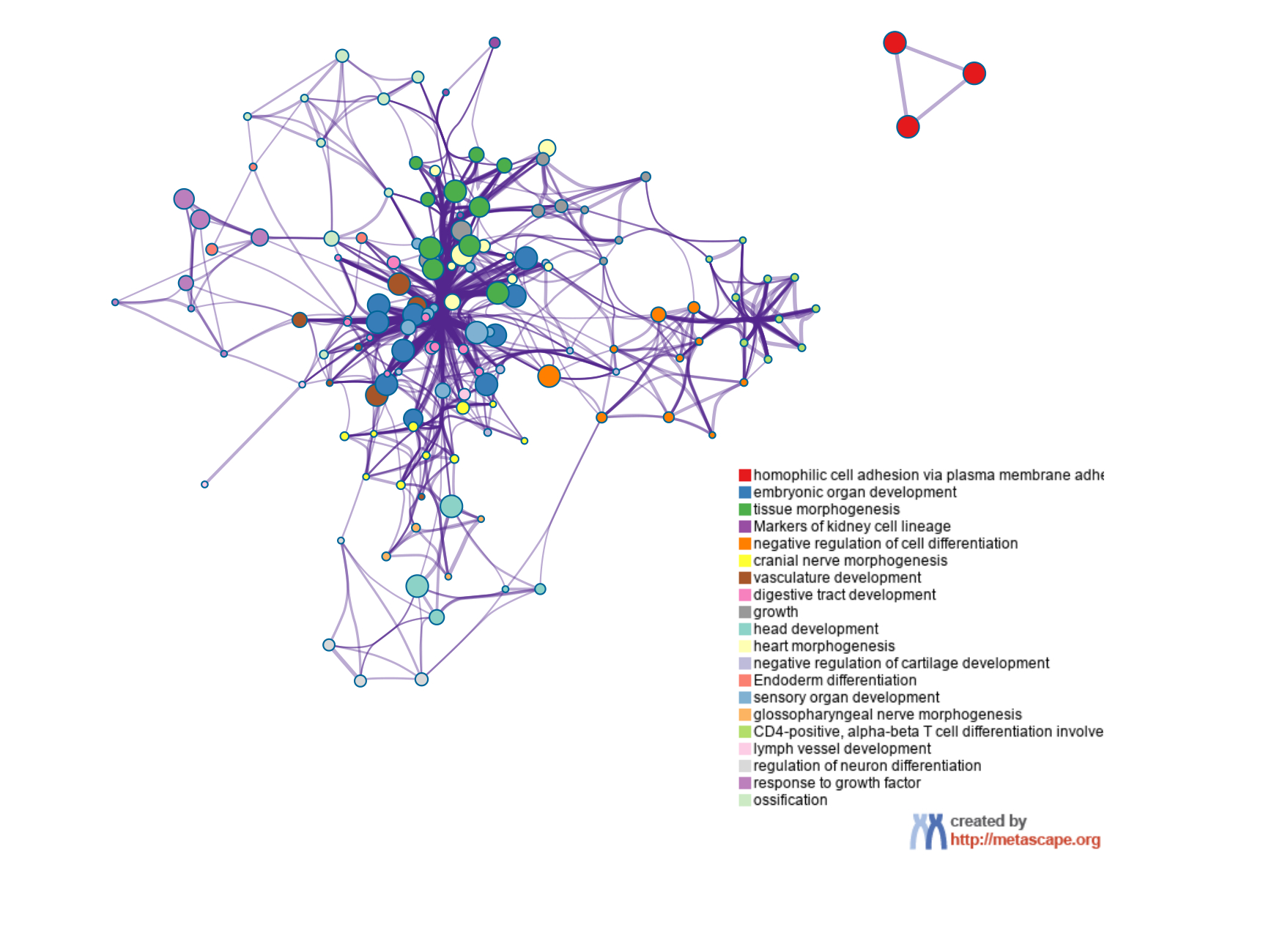

Supplement: Supplementary file 6 [file Image5.JPEG]

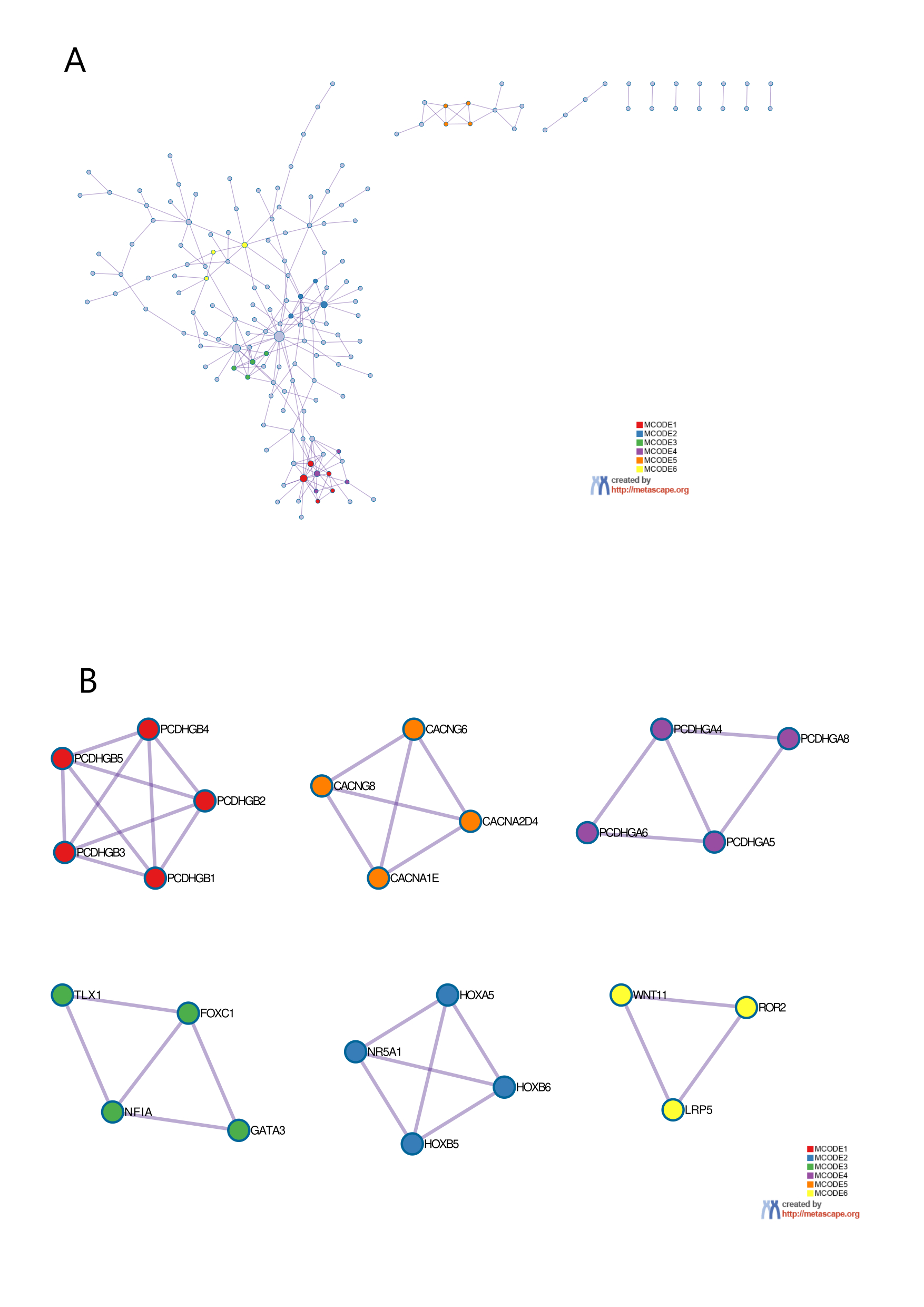

Supplement: Supplementary file 8 [file Image8.JPEG]

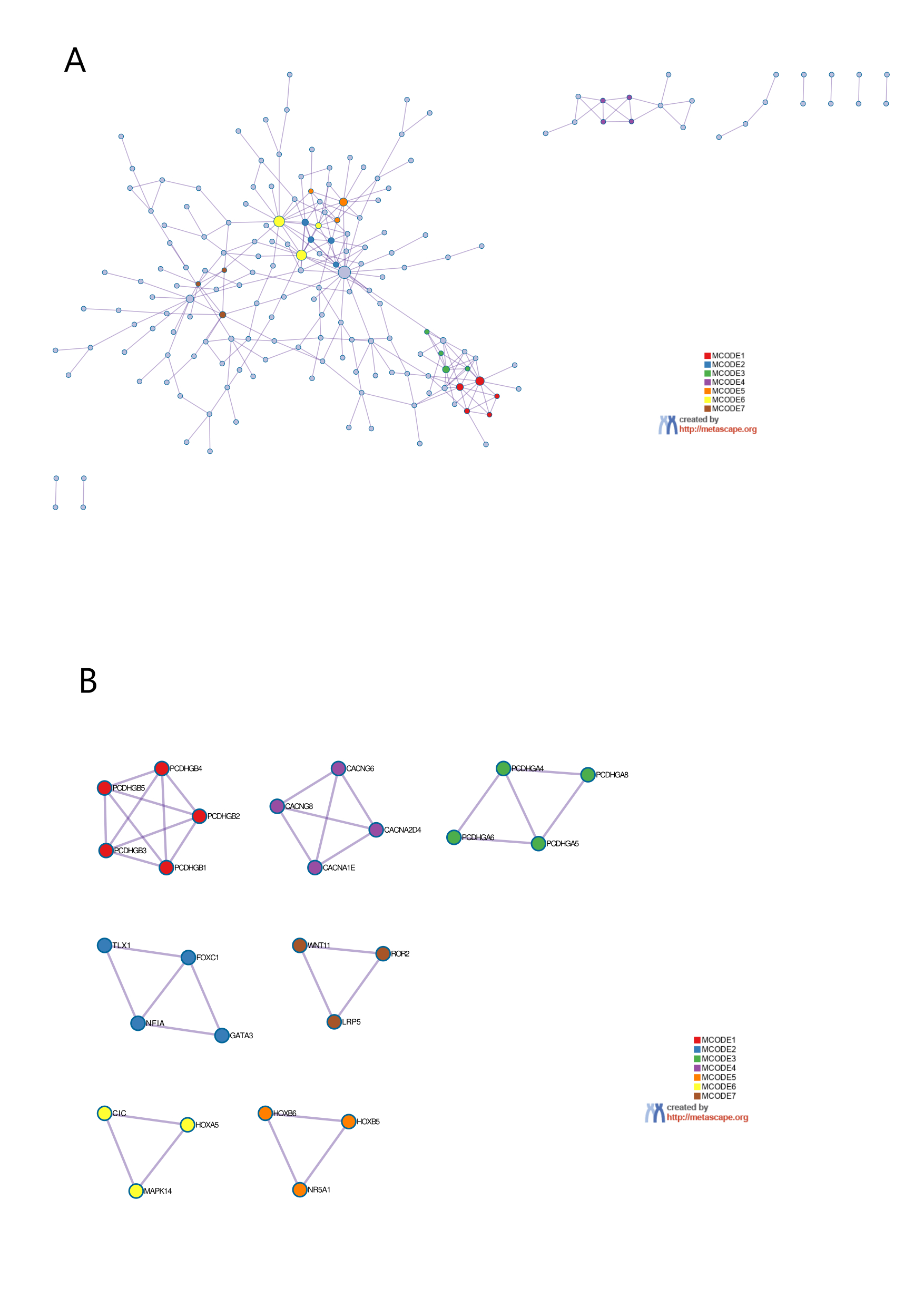

Supplement: Supplementary file 9 [file Image9.jpg]

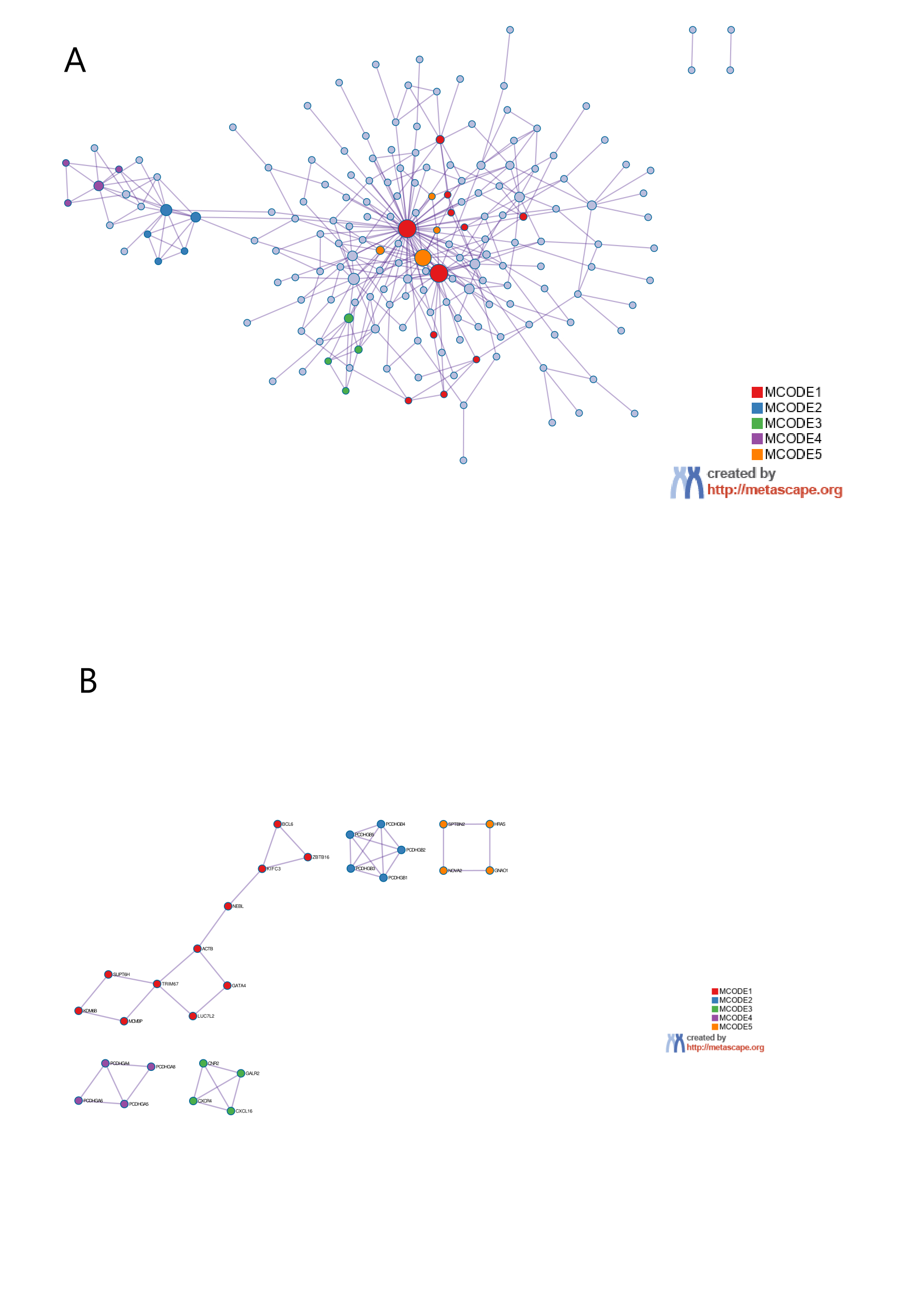

Supplement: Supplementary file 10 [file Image6.JPEG]
